# Berberine induces apoptosis and DNA damage in MG-63 human osteosarcoma cells

**DOI:** 10.3892/mmr.2014.2405

**Published:** 2014-07-21

**Authors:** YU ZHU, NAN MA, HUI-XIANG LI, LIN TIAN, YU-FENG BA, BIN HAO

**Affiliations:** 1 Department of Orthopedic Surgery, The Second Affiliated Hospital of Zhengzhou University, Zhengzhou, Henan 450014, P.R. China; 2 Department of Interventional Radiology, The First Affiliated Hospital of Zhengzhou University, Zhengzhou, Henan 450052, P.R. China; 3 Department of Pathology, The First Affiliated Hospital of Zhengzhou University, Zhengzhou, Henan 450052, P.R. China; 4 Department of Medicine, Zhengzhou Ninth People’s Hospital, Zhengzhou, Henan 450014, P.R. China; 5 Department of Thoracic Surgery, The Affiliated Tumor Hospital of Zhengzhou University (Henan Tumor Hospital), Zhengzhou, Henan 450008, P.R. China; 6 Department of Urology, The Second Affiliated Hospital of Zhengzhou University, Zhengzhou, Henan 450014, P.R. China

**Keywords:** berberine, DNA damage, apoptosis, γH2AX foci, DNA fragmentation

## Abstract

Berberine, an isoquinoline alkaloid extracted from the dry root of Coptidis Rhizoma, has been found to exhibit marked anticancer effects on a panel of established cancer cells. Among the human osteosarcoma lines treated, MG-63 cells were found to be the most sensitive. The present study investigated the potential genotoxic effect of berberine on MG-63 human osteosarcoma cells. The effect of berberine on cell viability was determined using a 3-(4,5-dimethylthiazol-2-yl)-2,5-diphenyltetrazolium bromide assay and cell apoptosis was analyzed by flow cytometry and a DNA ladder assay. γH2AX focus formation was used to detect DNA damage in MG-63 cells. Berberine induced a significant increase in apoptosis in MG-63 cells in a concentration- and time-dependent manner, as determined by DNA fragmentation analysis and flow cytometry. Furthermore, berberine induced significant concentration- and time-dependent increases in DNA damage compared with that in the negative control. In conclusion, these observations indicated that berberine induced apoptosis and DNA damage in MG-63 cells.

## Introduction

Osteosarcoma is a cancerous bone tumor that usually develops in adolescents (1). Significant improvements in patient survival rates have been achieved in recent years. Bioactive compounds derived from natural products have been used for thousands of years for therapy, pre-dating recorded history (2). Berberine, an isoquinoline alkaloid derived from the Chinese herb Huanglian, is used as a botanical drug (3). In China, berberine is commonly prescribed for the treatment of gastrointestinal complaints, diarrhea and other conditions. Accumulative evidence from *in vitro* studies has demonstrated that berberine possesses anticancer and anti-inflammatory activity in different types of human cancer cells, including osteosarcoma (4), epidermoid carcinoma (5), lung cancer (6), melanoma (7), prostate cancer (8) and liver cancer (9) cells. Animal studies have demonstrated that berberine is able to suppress chemical-induced carcinogenesis (10), tumor formation (11) and tumor invasion (12,13). Apoptosis and DNA damage have previously been revealed to be effective in eliminating cancer cells. Numerous natural compounds, including berberine, have been developed as preventive and treatment agents against cancer. However, to the best of our knowledge, few studies have examined the potential therapeutic effects of berberine in osteosarcoma.

The present study examined the effects of berberine on MG-63 cells in culture using DNA fragmentation analysis and flow cytometry. It has previously been demonstrated that the formation of DNA double-strand breaks induces γH2AX aggregations in nuclei, and it has been suggested that γH2AX focus formation is a sensitive method for detecting DNA double-strand breaks (14). A threshold of ≥4 γH2AX foci/cell has been found to be optimal for the determination of DNA damage (15). Thus the extent of DNA damage was observed in berberine-treated cells, as determined by measuring γH2AX focus formation.

## Materials and methods

### Drugs and materials

Berberine (purity, >98%) was purchased from Tianping Pharmaceutical Co. (Shanghai, China). The compound was dissolved in dimethyl sulfoxide (DMSO). N-methyl-N′-nitro-N-nitrosoguanidine (MNNG), normal-melting agarose, 4′,6-diamidino-2-phenylindole (DAPI), 3-(4,5-dimethylthiazol-2-yl)-2,5-diphenyltetrazolium bromide (MTT), Tween-20 and paraformaldehyde were obtained from Sigma Chemical Co. (Silicon Valley, CA, USA). The apoptosis detection kit was obtained from BD Pharmingen (San Diego, CA, USA). Triton X-100, fetal bovine serum (FBS), xylene cyanol and bromophenol blue were obtained from Sangon Biotech Shanghai Co., Ltd. (Shanghai, China). All other chemicals were purchased from Sinopharm Chemical Reagent Co., Ltd. (Shanghai, China).

### Cell culture

The MG-63 human osteosarcoma cell line (wild type) was purchased from the Cell Bank of Type Culture Collection of the Chinese Academy of Sciences (Shanghai, China). The cells were cultured in Dulbecco’s modified Eagle’s medium supplemented with 10% heat-inactivated FBS, penicillin (100 U/ml) and streptomycin (100 U/ml). The cells were incubated at 37°C in a 5% CO_2_ incubator. The medium was exchanged once every two days. Following treatment, the cells were harvested by trypsinization.

### Analysis of cytotoxicity

The cytotoxicity was determined using the MTT assay (16). MG-63 cells were seeded at a density of 1×10^4^ cells/well in 100 μl of cell culture medium and then placed in a 96-well plate. Following 12 h of incubation, the cells were treated with 0–80 μM berberine for 12 and 24 h. MTT solution (5 mg/ml) was then added to each well and the samples were incubated at 37°C for 4 h. Subsequently, the supernatant was removed and replaced with 100 μl DMSO. The optical density of the control and drug-treated wells was measured using an automated microplate reader (Multiskan Ex; Ani Lab systems Ltd., Vantaa, Finland) at a test wavelength of 570 nm.

### Flow cytometric analysis of berberine-induced apoptosis in MG-63 cells

To determine the externalization of phosphatidylserine by fluorescein isothiocyanate (FITC)-labeled Annexin V and propidium iodide (PI), flow cytometry was used as previously described (17). Briefly, the cells were treated with berberine at concentrations of 20, 40, 60 and 80 μM for 12 and 24 h. The cells were washed twice with cold phosphate-buffered saline (PBS) and resuspended in 500 μl binding buffer at a concentration of 1×10^6^ cells/ml. Then, 5 μl Annexin V-FITC solution and 5 μl PI (1 mg/ml) were added. The cells were incubated at 37°C for 30 min and analyzed by flow cytometry within 1 h. The number of apoptotic cells were counted and presented as a percentage of the total cell count.

### DNA extraction and detection of DNA fragmentation

The DNA ladder assay was performed as previously described (18), with slight modifications. After treating the cells with berberine and MNNG at concentrations of 50 and 20 μM, respectively, for 24 h, pellets containing 1×10^6^ cells were lysed in lysis buffer [10 mM Tris-HCl (pH 8.0), 25 mM ethylenediaminetetraacetic acid (EDTA), 0.5% sodium dodecyl sulfate, 100 mM NaCl and 400 g/ml protease K] for 120 min at 56°C and then treated with 10 mg/ml RNase A for an additional 50 min at 37°C. The lysates were centrifuged (12,000 × g for 30 min at 4°C) and the supernatant was collected. The fragmented DNA was extracted from the supernatant with a neutral phenol:chloroform:isoamyl alcohol mixture (v/v/v; 25:24:1). The DNA pellet was precipitated by adding isopropanol, washed with 75% ethanol and dissolved in Tris-EDTA buffer (10 mM Tris-HCl and 1 mM EDTA; pH 8.0). DNA fragmentation was detected by gel electrophoresis and the bands were stained with ethidium bromide for UV light visualization.

### γH2AX focus staining

The phosphorylation of histone H2AX (a marker of DNA double-strand breaks) was analyzed as previously described (15), with slight modifications. Briefly, 1×10^5^ cells were seeded into 6-well culture plates containing a glass cover slip in each well. Following treatment, the cells were fixed in 4% paraformaldehyde for 15 min, washed with PBS and permeabilized in 0.2% Triton X-100. Following inhibition with blocking serum for 1.5 h, the samples were incubated with a mouse monoclonal anti-H2AX antibody (1:1,000; Cell Signaling Technology, Inc., Boston, MA, USA) for 2 h, followed by incubation with FITC-conjugated goat anti-mouse secondary antibody (1:500; Cell Signaling Technology, Inc.) for 1 h. For staining the nuclei, DAPI was added to the cells and incubated for another 15 min. The cover slip was then removed from the plate, mounted on a glass slide and observed using an Olympus BX53 fluorescent microscope (Olympus, Tokyo, Japan).

### Statistical analysis

Data are expressed as the mean ± standard error of the mean of three independent experiments. The differences among the treated groups and the negative control were compared by one-way analysis of variance. The Newman-Keuls multiple comparisons test was applied. P<0.05 was considered to indicate a statistically significant difference. All statistical analyses were performed using SPSS 17.0 (SPSS, Inc., Chicago, IL, USA).

## Results

### Cytotoxic effect of berberine on MG-63 cells

**Figure 1:**
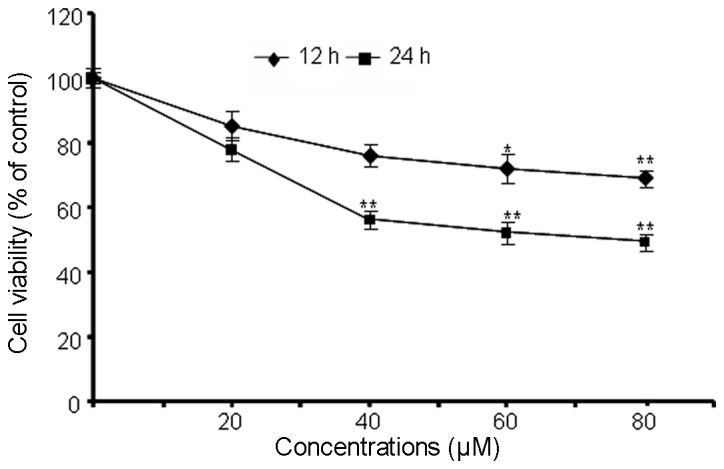
Cytotoxicity of berberine in MG-63 cells. The cells were treated for 12 and 24 h in the presence of berberine. Cell viability was then determined using a 3-(4,5-dimethylthiazol-2-yl)-2,5-diphenyltetrazolium bromide assay and expressed as the mean ± standard error of the mean of three separate experiments. ^*^P<0.05 and ^**^P<0.01, compared with the control.

The results of the trypan MTT assay demonstrated that berberine induced a concentration- and time-dependent decrease in the viability of MG-63 cells compared with the control (Fig. 1), indicating that berberine has cytotoxic effects on MG-63 cells.

### Berberine induced apoptosis in MG-63 cells

**Figure 2:**
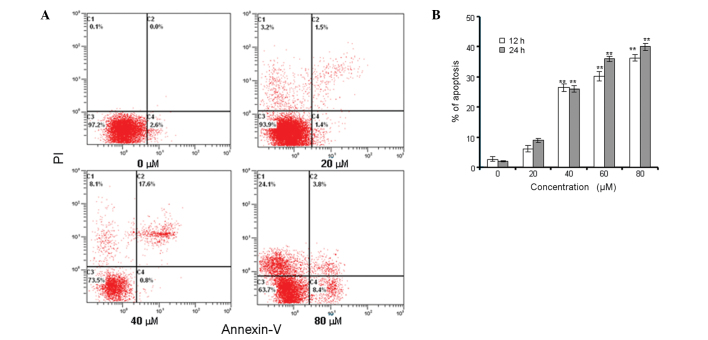
Berberine induces apoptosis in MG-63 cells. (A) Flow cytometric analysis of apoptosis using Annexin-V and PI double staining (12 h). (B) Apoptotic incidences of MG-63 cells treated with 0, 20, 40, 60 or 80 μM berberine for 12 and 24 h. ^*^P<0.05 and ^**^P<0.01, compared with control. PI, propidium iodide.

**Figure 3:**
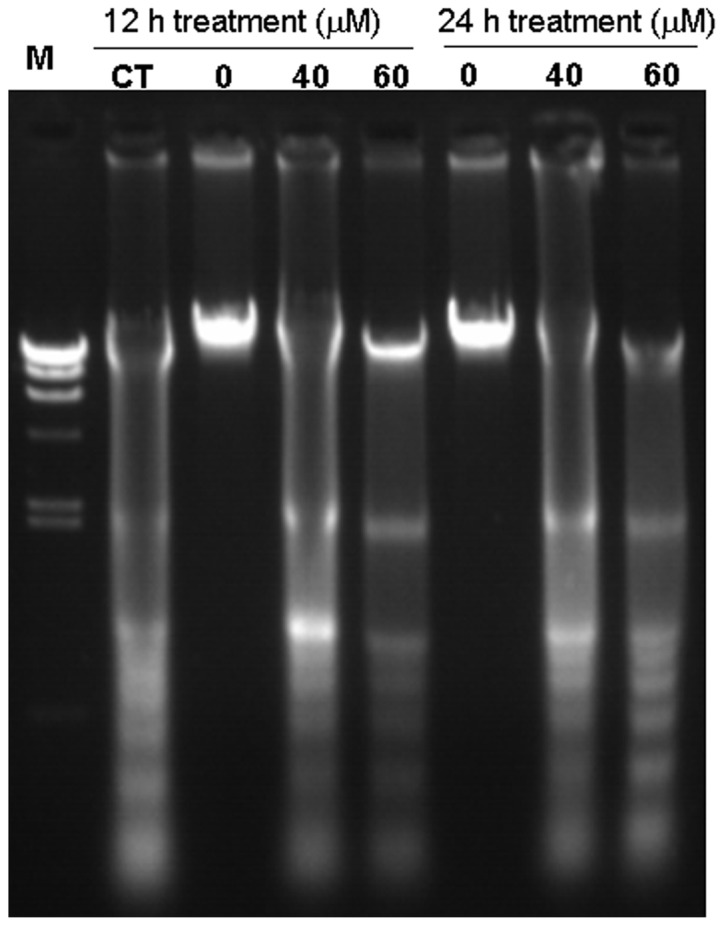
Berberine induces apoptosis-associated DNA fragmentation in MG-63 cells by the separation of urea polyacrylamide gel electrophoresis. M, marker (1 kb DNA ladder); CT, a positive control of methylnitronitrosoguanidine (20 mM).

Annexin V/PI staining was used to analyze whether the loss of cell viability induced by berberine was associated with apoptosis. Fig. 2 shows the rate of cell apoptosis detected by double-labeling flow cytometry with Annexin V and PI. A concentration- and time-dependent increase was observed in the apoptotic rate of MG-63 cells exposed to berberine. The apoptotic rates of MG-63 cells treated with berberine at 20, 40, 60 and 80 μM increased to 6.1±1.1, 26.5±1.3, 30.2±2.8 and 36.3±1.0% following 12 h; 9.0±0.7, 26.1±1.5, 36.4±1.8 and 40.0±1.2% following 24 h, respectively. By contrast, the control cells showed apoptosis rates of only 2.8±0.8 and 2.0±0.2% following 12 and 24 h, respectively (Fig. 2B). Furthermore, it was revealed that berberine induced significant DNA fragmentation in MG-63 cells (Fig. 3). DNA fragmentation was observed in cells treated with berberine at 40 and 60 μM following 12 and 24 h. These results suggest that the anticancer activity of berberine involves the induction of apoptosis.

### γH2AX foci show DNA double-strand breaks are induced by berberine

**Figure 4:**
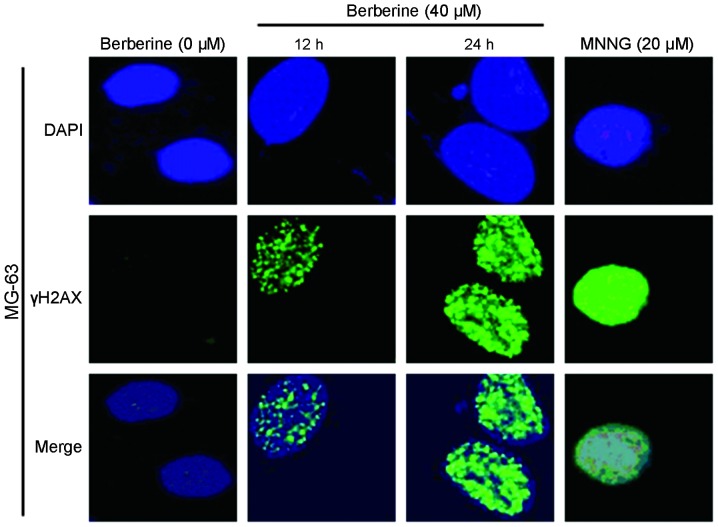
DNA double-strand breaks as illustrated by γH2AX foci formation in MG-63 cells. Anti-γH2AX monoclonal antibody was used to detect DNA damage foci immunofluorescence and DAPI was used to stain nuclei (magnification, ×400). DAPI, 4′,6-diamidino-2-phenylindole; MNNG, methylnitronitrosoguanidine.

**Figure 5:**
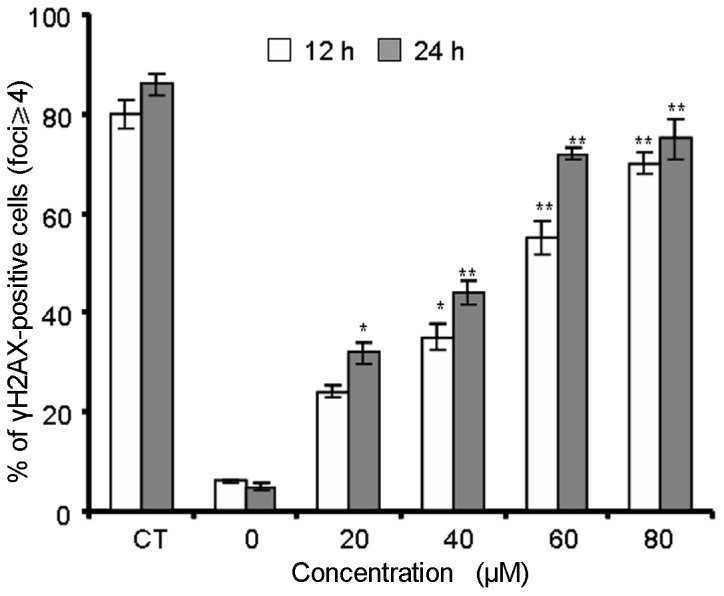
Percentage of γH2AX-positive MG-63 cells treated with 20 mM methylnitronitrosoguanidine (CT) and 0, 20, 40, 60 or 80 mM berberine for 12 and 24 h. Data are presented as the mean ± standard error of the mean of three independent experiments. ^*^P<0.05 and ^**^P<0.01 compared with control

The immunofluorescent images of histone H2AX phosphorylation in γH2AX-stained MG-63 cells are shown in Fig. 4. Treatment with berberine resulted in time-dependent induction of γH2AX foci. In the control group, MG-63 cells had few γH2AX foci in the nuclei, with only ~6% of cells containing more than four foci (Fig. 5). Berberine and MNNG treatment induced foci formation and increased the percentage of γH2AX-positive cells. The data in Fig. 5 show that berberine and MNNG exhibited distinct concentration- and time-dependent effects (P<0.01) on γH2AX foci formation in MG-63 cells.

## Discussion

Over the past decade, interest in the pharmacological effects of bioactive compounds with respect to use in cancer treatments and for cancer prevention has markedly increased (19). Accumulating evidence has demonstrated a correlation between natural compounds and cancer prevention (5,6,9,20). Thus, evaluation of ancient medicinal herbs may provide the basis for the development of chemopreventive methods and strategies. Berberine was used widely in ancient therapeutic medicinal practices (3). It has been demonstrated to exert numerous anticancer activities in various types of cancer cells through different cytotoxic effects (21). Previous studies have demonstrated that human osteosarcoma cells (U2OS, Saos-2 and HOS) treated with berberine exhibited cell cycle arrest and apoptosis (4).

The present study found that berberine (20–80 μM) inhibited growth of MG-63 cancer cells through induction of apoptosis and DNA damage *in vitro*. Furthermore, berberine was demonstrated to induce apoptosis and DNA damage of MG-63 cells in a dose- and time-dependent manner. Therefore, berberine acts as a potent genotoxin by inducing marked accumulation of DNA double-strand breaks. The results indicated that treatment with berberine triggered a cascade that includes DNA damage (Fig. 4).

Berberine induced double-strand DNA damage and apoptosis in Ehrlich ascites carcinoma cells (20). Treatment with berberine *in vivo* resulted in additive cytotoxicity and indicated that berberine has potent antitumor activity against human and rat malignant brain tumors (22). A study involving *Saccharomyces cerevisiae* demonstrated that berberine exhibited no cytotoxic, mutagenic or recombinogenic activity in non-dividing cells. However, it had significant cytotoxic and cytostatic effects on dividing cells (23). Notably, berberine is more toxic to yeast mutants that are deficient in rad52-1, suggesting that homologous recombination repair is required for the repair of berberine-induced DNA damage. These results suggest that berberine possesses recombinogenic activity. By contrast, coralyne, a close derivative of berberine, has not been revealed to have detectable mutagenic activity, when analyzed using the Ames test (24). Further studies are required to evaluate the mutagenic activities of berberine.

Berberine has been widely prescribed for the treatment of bacterial diarrhea and has potential applications in several other diseases, including cancer. The findings of the present study demonstrated that berberine causes DNA damage in cultured cells, which raises concerns for its safety in clinical use. However, the present study did not determine whether the induction of apoptosis is directly associated with the genotoxicity of berberine. The mechanisms underlying the genotoxicity of berberine require further investigation to clearly demonstrate that berberine causes genotoxocity and apoptosis. Furthermore, more studies are required to understand the biological consequences of DNA damage on exposure to berberine *in vivo*. Considering the widespread clinical use of berberine, thorough evaluation of its genotoxocity *in vivo* is warranted.
